# The prognostic value of the Charlson comorbidity index in aged patients with intracerebral hemorrhage

**DOI:** 10.1186/s12883-022-02980-z

**Published:** 2022-11-28

**Authors:** Tianjie Zhang, Ruiqi Chen, Dingke Wen, Xing Wang, Lu Ma

**Affiliations:** 1 West China School of medicine, West China Hospital of Sichuan University, Sichuan Province, Chengdu, 610041 China; 2 Department of Neurosurgery, West China Hospital of Sichuan University, Chengdu, 610041 Sichuan Province China

**Keywords:** Intracerebral hemorrhage, Charlson comorbidity index, Old patients, Stroke outcome, Comorbidity

## Abstract

**Background:**

Comorbidities are common in aged intracerebral hemorrhage patients. The purpose of this study was to assess whether the Charlson Comorbidity Index (CCI) was associated with in-hospital death and short-term functional outcome in elderly patients (age ≥ 70) with intracerebral hemorrhage (ICH).

**Methods:**

This was a retrospective cohort of aged ICH patients (≥70 years old) admitted within 24 hours of ICH onset. The CCI was derived using hospital discharge ICD-9 CM codes and patient history obtained from standardized case report forms. Multivariable logistic regression was used to determine the independent effect of the CCI score on clinical outcomes.

**Results:**

In this cohort of 248 aged ICH patients, comorbid conditions were common, with CCI scores ranging from 2 to 12. Logistic regression showed that the CCI score was independently predictive of 1-month functional outcome (OR = 1.642, *P* < 0.001) and in-hospital death (OR = 1.480, *P* = 0.003). Neither ICH volume nor the presence of IVH was an independent predictive factor for 1-month functional outcome or in-hospital mortality (*P* < 0.05).

**Conclusion:**

Comorbid medical conditions as assessed by the CCI independently influence short-term outcomes in aged ICH patients. The characteristics of the hematoma itself, such as ICH volume and the presence of IVH, seem to have a reduced effect on it.

## Introduction

Intracerebral hemorrhage (ICH) accounts for 6.5 to 19.6% of all strokes [1, 2], and it is significantly more common in the elderly population [3], accounting for approximately 25% of all strokes [4]. As the population continues to age, the prevalence of ICH in increasingly aged patients will increase accordingly [5]. Despite numerous advances in stroke management and neurocritical care, ICH remains the most devastating cerebrovascular disease subtype with significant rates of disability and mortality. The 1-year survival from ICH is approximately 40%, and older age is associated with an increased risk of 30-day death [3, 6].

Over the past few decades, great advances have been made in neurocritical care that may help improve the outcome of cerebral hemorrhage. For these aged ICH patients, stratification is of great necessity to appropriately allocate limited medical resources and counsel both patients and their families in goals of cure. However, considering the high morbidity and mortality of ICH in the elderly population and the heavy burden it may impose on families and society, it is vital that these decisions are based on accurate and repeatable information. Therefore, it is essential to reevaluate previously validated scores, such as the Charlson Comorbidity Index (CCI), to assess whether their prognostic ability can improve with medical advances [7].

Comorbidities are common in ICH patients, especially in elderly individuals, [5, 8] and they are widely considered as one of the factors affecting the outcomes of stroke [9–13], but few studies have focused on hemorrhagic stroke. The CCI accounts for multiple comorbidities by creating a sum score weighted according to the presence of various conditions, which was originally developed against 1-year death, and its validity as a predictor of other outcome measures, either in the short term or in-hospital death, has not been tested in ICH. Therefore, the aim of this study was to determine whether a patient’s prehemorrhage medical comorbidities, as assessed by the CCI, affect ICH short-term outcomes. The results of this study may be helpful to obtain a better understanding of ICH in the elderly age group and to assist in the choice of best clinical decision making.

## Methods

### Materials and patients

We performed a retrospective chart review of aged ICH patients (≥70 years old) admitted to the Department of Neurosurgery, West China Hospital (WCH), from December 2018 to December 2020. The study was approved by the Institutional Review Board (IRB) of WCH for retrospective chart review without the need for patient consent. The inclusion criteria were as follows: (1) over 70 years of age; (2) admitted to our department within 24 hours of ICH onset; (3) admission diagnosis of ICH based on brain CT scans; (4) available clinical data, including medical history and baseline information; and (5) neuroimaging to evaluate the characteristics of the hematoma. The exclusion criteria were as follows: (1) secondary ICH (aneurysm, vascular malformation, or tumor); (2) unavailability of outcome data; and (3) patients who declined to participate.

### CCI

The CCI incorporates the use of 19 separate health conditions on a 1- to 6-point scale (minimum score 0, maximum score 37) to develop a composite score [14]. For example, “diabetes with end-organ damage” and “hemiplegia” are each assigned 2 points within the CCI scoring system. The specific content of CCI score is based on ICD-9 code [15]. Variables in the CCI were retrospectively collected from the hospital information system (HIS) of WCH. The CCI was retrospectively calculated at admission on the basis of International Classification of Diseases, Ninth Revision, Clinical Modification codes. Two experienced, uninformed researchers independently scored and took the final average. More diseases appeared during hospitalization, except for cerebrovascular disease and hemiplegia secondary to ICH.

### Data collection and grouping

For every patient, the variables collected included age, sex, height, weight, clinical record, previous medical history, anticoagulant (AC) use, baseline CT imaging characteristics and admission Glasgow Coma Scale (GCS). All initial computed tomography (CT) scans of the head were reviewed for the location and presence of IVH, subarachnoid hemorrhage (SAH) and finger-like projection. ICH volume was calculated using the ABC/2 method [16]. All the data were obtained from the hospital information system (HIS) of WCH except for the CCI scores, which were assessed and calculated by an experienced neurosurgeon. In addition, research assistants collected the outcomes of every patient for the follow-up period, including the 30-day modified Rankin scale (mRS) score and in-hospital death.

### Outcome measures

Patients were grouped according to the modified Rankin Scale (mRS) score at 30 days from onset. The mRS was measured at the outpatient visit or by telephone using a structured interview [17]. Patients with a 30-day mRS score ≥ 3 points were divided into the poor-outcome group, and patients with a 30-day mRS score < 3 points were divided into the favorable-outcome group. We first compared the differences in the CCI and other indicators between the two groups by univariate analysis. After that, the results with *P* < 0.1 and other possible indicators associated with 30-day prognosis and death were included in a multivariate analysis to explore independent factors associated with poor prognosis and in-hospital death in elderly ICH patients. Finally, the integrity and completeness of all data were regularly checked by another experienced researcher.

### Statistical analyses

All statistical analyses were performed by SPSS statistical software (version 22.0; SPSS Inc., Chicago, Illinois, USA) and MedCalc statistical software (version 15.2; MedCalc Software, Mariakerke, Ostend, Belgium). Continuous data are expressed as the mean ± standard deviation (SD). Categorical parameters are reported as frequencies and percentages. First, the correlation between variables was analyzed by univariate analysis. Chi-square test is performed on categorical and binary data, and Student’s t test is used for continuous variables. The relationship between the unadjusted CCI score and 1-month functional status after the event as well as in-hospital death were determined. Finally, the independent effect of CCI score on each outcome was then determined by multivariable logistic regression, and sex, IVH, ICH location admission GCS and ICH volume were controlled. Two-tailed *p* < 0.05 was considered significant.

## Results

A total of 248 patients were analyzed in the study. Table 1 shows the frequency of CCI categories in our ICH cohort. All our samples are older than 70 years old, of which 28.2% are over 80 years old. The most frequent comorbidities were pulmonary disease found in 80.6% of patients, followed by cerebrovascular disease (62.5%) and diabetes (15.7%, without complications in 13.7%). Other frequent conditions were congestive heart failure (6.5%), ulcer disease (5.6%), and hemiplegia (5.6%).

**Table 1:** Frequency of Charlson Comorbidity Index categories in the intracerebral hemorrhage cohort (*n* = 248)

Condition	Frequency, N (%)
Age	
70-79 (%)	178 (71.8)
80-89 (%)	64 (25.8)
90-95 (%)	6 (2.4)
Pulmonary disease (%)	200 (80.6)
Cerebrovascular disease (%)	155 (62.5)
Diabetes without end-organ damage (%)	34 (13.7)
Congestive heart failure (%)	16 (6.5)
Ulcer disease (%)	14 (5.6)
Hemiplegia (%)	14 (5.6)
Dementia (%)	11 (4.4)
Any tumor (%)	8 (3.2)
Mild liver disease (%)	5 (2.0)
Moderate or severe renal disease (%)	5 (2.0)
Diabetes with end-organ damage (%)	5 (2.0)
Moderate or severe liver disease (%)	5 (2.0)
Peripheral vascular disease (%)	4 (1.6)
Myocardial infarction (%)	3 (1.2)
Connective tissue disease (%)	2 (0.8)
Metastatic solid tumor (%)	2 (0.8)
Leukemia (%)	1 (0.4)
Lymphoma (%)	0 (0.0)
AIDS (%)	0 (0.0)

**Fig. 1:**
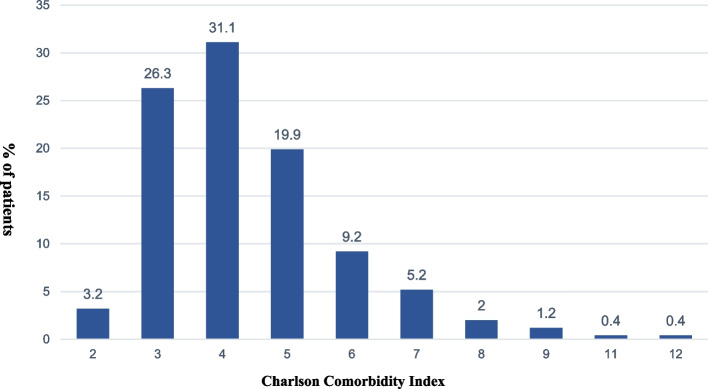
Frequencies of the Charlson comorbidity index scores across the entire aged ICH cohort (*n* = 248). The X-axis is the CCI score, and the Y-axis is the proportion of patients

Figure 1 gives the distribution of CCI scores. The mean CCI score was 4.41 (standard deviation 1.54), with a median of 4 (25th to 75th percentile: 3-5). The range was 2-12.

Overall, 17 patients (8%) died during hospitalization, and 127 patients (51.2%) had a poor outcome after 30 days of ICH. Table 2 shows the distribution of the demographic and clinical characteristics of the study participants who were grouped into poor or favorable outcomes based on the 30-day mRS score. The poor outcome group included higher CCI scores [(4.93 ± 1.57) vs (3.92 ± 1.33), *p* < 0.001], lower admission GCS [(8.23 ± 3.32) vs. (13.48 ± 1.68), *p* < 0.001], higher ICH volume [(58.62 ± 47.20) vs. (24.32 ± 21.52), *p* < 0.001], and a higher proportion of IVH (72.7 vs. 34.6%, *p* < 0.001). SAH (41.5 vs. 22.2%, *p* = 0.032) and finger-like projection (64.2 vs. 24.1%, *p* < 0.001) rates were higher in the poor outcome group in aged lobar hemorrhage patients.

**Table 2:** Demographic and clinical characteristics of elderly SICH patients

	Total (*n* = 248)	Poor Outcome (*n* = 121)	Favorable Outcome (*n* = 127)	P
Men (%)	152 (61.3)	68 (56.2)	84 (66.1)	0.171
Weight, kg (mean ± SD)	59.14 ± 10.78	58.03 ± 11.42	60.05 ± 10.21	0.287
Hight, cm (mean ± SD)	161.52 ± 8.02	160.67 ± 7.45	162.26 ± 8.46	0.233
Smoke (%)	41 (16.5)	17 (14.0)	24 (18.9)	0.304
Alcohol (%)	33 (13.3)	13 (10.7)	20 (15.7)	0.246
Anticoagulant agent used (%)	8 (3.2)	5 (4.1)	3 (2.4)	0.430
Hypertension (%)	199 (80.2)	99 (81.8)	100 (78.7)	0.543
CCI, (mean ± SD)	4.42 ± 1.54	4.93 ± 1.57	3.92 ± 1.33	< 0.001
Admission GCS, (mean ± SD)	10.92 ± 3.70	8.23 ± 3.32	13.48 ± 1.68	< 0.001
Hematoma Location				
Infratentorial (%)	34 (13.7)	19 (15.7)	15 (11.8)	0.373
Supratentorial (%)	214 (86.3)	102 (84.3)	112 (88.2)	
Lobar (%)	107 (43.1)	53 (43.8)	54 (42.5)	0.839
SAH, % (n)	34 (31.8)	22 (41.5)	12 (22.2)	0.032
Finger-like Projections (%)	47 (43.9)	34 (64.2)	13 (24.1)	< 0.001
ICH Volume, cm^3^, (mean ± SD)	41.06 ± 40.16	58.62 ± 47.20	24.32 ± 21.52	< 0.001
IVH (%)	132 (53.2)	88 (72.7)	44 (34.6)	< 0.001

In multivariate analysis, as shown in Table 3, the CCI score (odds ratio (OR) 1.642, 95% CI 1.254 ~ 2.150, *P* < 0.001), admission GCS (OR 0.505, 95% CI 0.408 ~ 0.626, *P* < 0.001), and ICH location in infratentorial region (OR 3.492, 95% CI 1.489 ~ 5.690, *P* = 0.002) independently predicted 30-day mRS outcome. The CCI score (OR 1.408, 95% CI 1.147 ~ 1.910, *P* = 0.003), admission GCS score (OR 0.818, 95% CI 0.720 ~ 0.930, *P* = 0.002) and ICH location in infratentorial region (OR 5.058, 95% CI 1.200 ~ 10.305, *P* = 0.022) independently predicted in-hospital mortality.

**Table 3:** Multivariate analysis of 30-days functional outcome and in-hospital mortality

		30-day mRS outcome		In-hospital mortality	
		OR (95% CI)	*P* Value	OR (95% CI)	*P* Value
CCI		1.642 (1.254 ~ 2.150)	< 0.001	1.480 (1.147 ~ 1.910)	0.003
ICH Volume		1.118 (0.904 ~ 1.533)	0.215	1.008 (0.997 ~ 1.018)	0.146
Admission GCS		0.505 (0.408 ~ 0.626)	< 0.001	0.818 (0.720 ~ 0.930)	0.002
ICH location	Supratentorial	Ref		Ref	
	Infratentorial	3.492 (1.489 ~ 5.690)	0.002	5.058 (1.200 ~ 10.305)	0.022
IVH	No	Ref		Ref	
	Yes	1.269 (0.558 ~ 2.885)	0.569	1.689 (0.623 ~ 4.579)	0.303
Sex	Male	Ref		Ref	
	Female	0.901 (0.386 ~ 2.102)	0.809	1.770 (0.779 ~ 4.022)	0.173

## Discussion

In our study, 248 elderly ICH patients aged over 70 years were retrospectively enrolled, and the results showed that the CCI score was independently associated with poorer short-term functional outcome and higher in-hospital mortality after adjusting for the components of the ICH score and sex.

Comorbidities, as measured by the CCI, are widely considered one of the factors affecting the outcomes of stroke [9–12], but few studies have focused on hemorrhagic stroke. Bar et al. found that comorbid medical conditions, as measured by the CCI, independently affect functional outcomes at 12 months after ICH [12]. However, Bar et al. investigated the age of patients over a wide range instead of a limited range. The comorbidity pattern in older ICH patients can be very different from that in younger patients, both in terms of the number and type of conditions. Compared with previous studies, we found that the incidence of pulmonary disease (80.6% vs. 11.1%) and cerebrovascular disease (62.5% vs. 23.5%) was particularly high in the elderly population. In addition, pulmonary [18–20] and cerebrovascular [21, 22] diseases have been proven to play an important role in the prognosis of ICH patients. A previous study focused on functional outcome after 1 year of ICH [12], and our study demonstrates that CCI, as a sum score weighted according to the presence of various comorbidities, also has an impact on the short-term prognosis of elderly ICH patients. Therefore, we consider that more intensive care and medical resources might be needed to improve the prognosis of elderly patients with higher CCI scores.

**Fig. 2:**
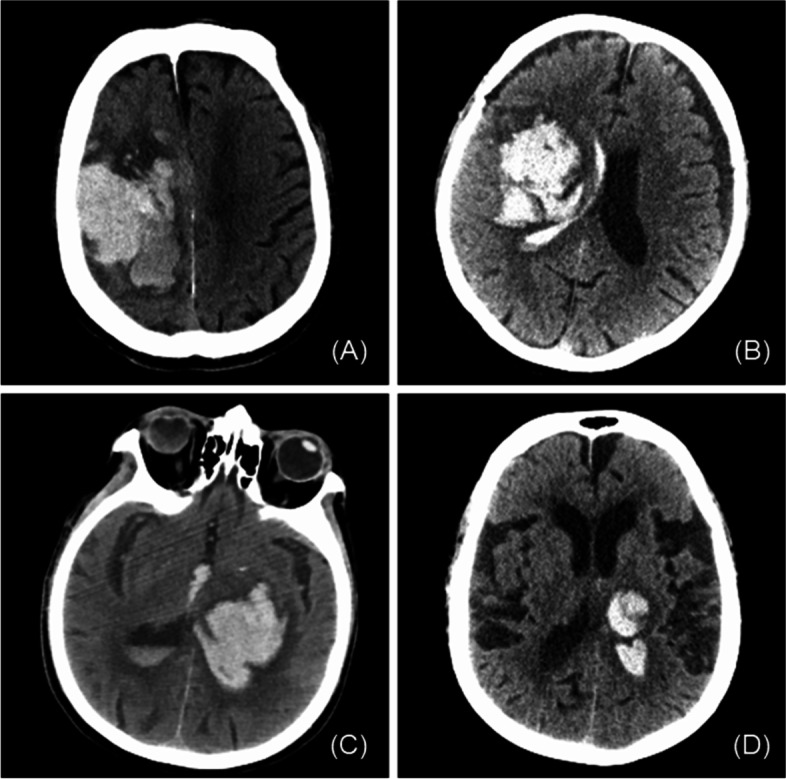
ICH in aged brain atrophy patients. There was no significant increase in intracranial pressure and no significant midline deviation in these patients with large hematoma or IVH

At the same time, our study found that neither IVH nor hematoma volume was an independent factor affecting the prognosis of elderly ICH patients. However, as components of the ICH score, ICH volume and IVH have been demonstrated to be independent predictors of prognosis in ICH patients [23, 24]. One possible explanation is that brain atrophy is more common in elderly individuals, which leaves more room for compensation in the brain (Fig. 2). Neither hematoma volume nor IVH has a significant impact on intracranial pressure in elderly patients and thus has a small impact on patient prognosis. At the same time, as a result of “immunosenescence”, elderly patients will experience an overall decline in the protective immune response [25], and secondary brain damage from hematoma may also be reduced. In addition, lobar intracerebral hemorrhage in elderly patients is most often caused by cerebral amyloid angiopathy (CAA) [26]. In our study, the proportion of patients with lobar hemorrhage was 43.1%. According to the Edinburgh criteria, 22.4% of them were high-risk CAA-related ICH patients, and 30.8% were moderate risk [27]. CAA is a common small vascular disease of the brain caused by progressive deposition of amyloid beta protein in the pia meningeal and cortical arterial walls and cortical capillaries. Patients with CAA-related ICH have a lower mortality rate than those with cerebral hemorrhage from other causes [28]. In previous clinical studies on the prognosis of ICH, CAA-related ICH has not been distinguished from hypertension-related intracerebral hemorrhage. As CAA-related ICH accounts for a high proportion in elderly patients, the prognostic factors traditionally associated with ICH may not be applicable to our elderly CAA-related ICH.

Our study has several limitations. First, as a retrospective study, a potential source of selection bias lies within the patient population itself. Second, several CCI factors, such as peripheral vascular disease and ulcer disease, may have been omitted, as they may not be routinely collected in the emergent setting and are difficult to assess in the routine daily medical exam. In addition, it is significant that subconscious bias towards elderly sicker patients may lead to less aggressive care. For ICH patients with high CCI scores, families may have lower treatment expectations and physicians may be more conservative in their treatment.

## Conclusion

Overall, for elderly patients with ICH, the CCI score, reflecting the presence of various comorbidities, might be an independent factor related to the patients’ short-term outcomes in terms of in-hospital mortality and 30-day prognosis, while the characteristics of the hematoma itself, such as ICH volume and presence of IVH, seem to have a reduced effect on patient short-term outcomes.

## Data Availability

The datasets used and/or analysed during the current study are available from the corresponding author on reasonable request.
